# Safety and efficacy of a novel retrograde route for femoral bone graft harvesting by Reamer-Irrigator-Aspirator: a pilot study on 24 patients

**DOI:** 10.1186/s13037-021-00315-4

**Published:** 2022-01-07

**Authors:** Fernando Bidolegui, Sebastián Pereira, Cristina Irigoyen, Robinson Esteves Pires

**Affiliations:** 1 Servicio de Ortopedia Y Traumatología, Hospital Sirio-Libanes, ECICARO, Ciudad Autónoma de Buenos Aires, Argentina; 2 Departamento Do Aparelho Locomotor, Universidade Federal de Minas Gerais, Belo Horizonte, MG Brazil; 3 Serviço de Ortopedia E Traumatologia, Instituto Orizonti, Belo Horizonte, MG Brazil

**Keywords:** Reamer-Irrigator-Aspirator, Bone graft, Bone defects, Osteomyelitis, Patient safety

## Abstract

**Background:**

The Reamer–Irrigator–Aspirator system was initially developed to reduce fat embolism and thermic necrosis during reamed intramedullary nail fixation of femoral shaft fractures. Currently, this system is used in extended applications including accessing large volume of autologous bone graft, as alternative for iliac crest harvesting. Antegrade femoral bone graft harvesting using the Reamer-Irrigator-Aspirator system is considered the standard technique. The aim of our study is to evaluate the efficacy (bone graft volume) and the complications (blood loss, postoperative pain, and incidence of iatrogenic fractures) of the Reamer–Irrigator–Aspirator system through the retrograde femoral route in a series of patients with post-traumatic bone defects or nonunions.

**Methods:**

A non-controlled single center retrospective observational cohort study was conducted in a level1 trauma center to evaluate all patients who were treated using the RIA system. Between November 2015 and May 2019, 24 patients (8 women and 16 men; mean age: 41 years [range 27–55 years]) with bone defects or nonunions underwent bone graft harvesting using the Reamer–Irrigator–Aspirator system through retrograde femoral route. Postoperative pain, complications, and bone graft volume were analyzed. Inclusion criteria was patients older than 18 years with a diagnosis of post-traumatic bone defect or associated tibial or femoral nonunion, with minimum 6-months follow, treated using the RIA. We hypothesized that the retrograde route of the RIA system is a safe and efficacious method for bone harvesting.

**Results:**

The average volume of collected graft was 45 cc (range 30–60 cc). In 83% of the cases, bone grafting was sufficient, while in 17% it was necessary to add iliac crest bone graft to completely fill the bone defect. A mean drop in postoperative hemoglobin of 4.1 g / dL (range 0.5–6.0 g / dL) was evidenced. In 4 cases (33%), a unit of packed red blood cells was required. Regarding postoperative pain, visual analogue scale after 3 months postoperatively was 1.6 in average. After 6 months, the value has decreased to 0.4. There were no perioperative or postoperative complications at 6-month follow-up.

**Conclusion:**

In this limited case series, large volumes of bone graft were harvested using the retrograde route of the RIA system and there were no intra-/ postoperative complications observed at 6-month follow-up. Therefore this novel technique appears safe and efficacious.

However, it’s important to highlight that future prospective controlled studies are necessary to validate the insights from this pilot study.

## Introduction

The Reamer–Irrigator–Aspirator (RIA; Synthes, West Chester, PA), initially developed to decrease thermal necrosis and the incidence of fat embolism during diaphyseal femur reaming, is also routinely used for the treatment of long bone osteomyelitis, since it removes infected and necrotic bone from the medullary canal. However, especially in the last decade, it has become a helpful alternative to the classic iliac crest bone graft harvesting, since it offers a larger volume of bone graft without the drawback of severe postoperative pain, which frequently accompanies the iliac crest bone graft harvesting [1–4]. Extended applications of the RIA system are clearing the femoral/tibial canal of cement debris and intramedullary nailing of long bones with metastatic diseases, since it potentially diminishes the tumor burden into the surrounding soft tissues and systemic circulation [5].

The standard donor site for the RIA system is the femur, mostly using the antegrade pathway [3].

Although a previous study comparing antegrade and retrograde techniques using the RIA system have shown similar morbidity and amount of bone graft harvested, a special concern exists when using the antegrade pathway due to the risk of eccentric reaming, potentially predisposing to an iatrogenic fracture [6].

Thus, the aim of this study was to evaluate the efficacy (bone graft volume) and safety (blood loss, postoperative pain, and incidence of iatrogenic fracture) of the RIA system through the retrograde route in a case series of patients with bone defects.

## Methods

A non-controlled single center retrospective observational cohort study was conducted in a level 1 trauma center to evaluate all patients who were treated using the RIA system between November 2015 and May 2019. The digitized medical records of the institution were used for data collection. All patients older than 18 years with a diagnosis of post-traumatic bone defect or associated tibial or femoral nonunion, with minimum 6-months follow, were included.

**Fig. 1:**
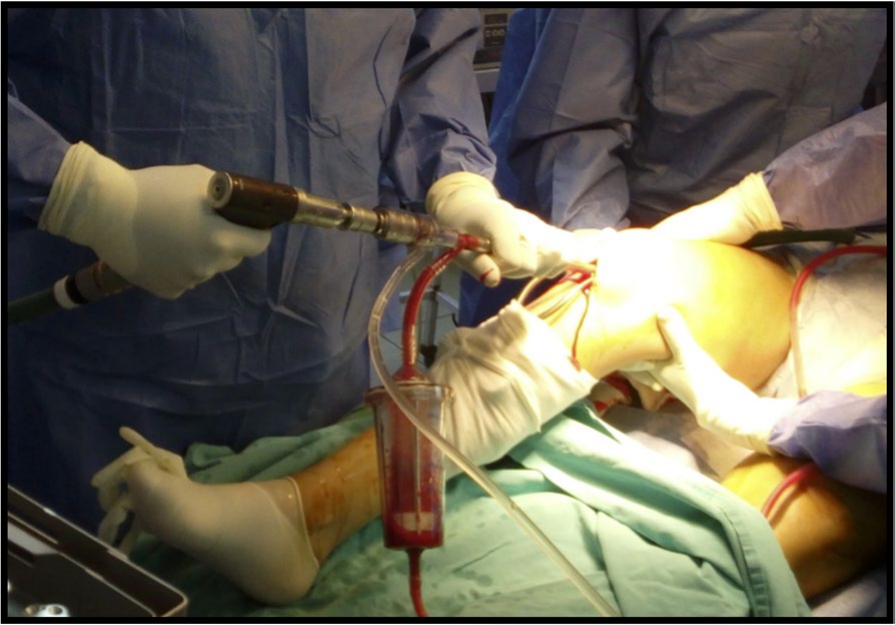
Intraoperative photograph depicting the retrograde pathway for bone graft harvesting using the RIA system

Figure 1 shows the retrograde portal for bone graft harvesting using the RIA system (Fig. 1). Those patients in whom the RIA system was used through an antegrade route in the femur (n: 2) or tibia (n: 1) and those who did not comply with the minimum follow-up (n: 1) were excluded.

The series consisted of 24 patients (8 women and 16 men). The mean age was 41 years (range 27–55 years). The diagnoses were: 10 aseptic nonunions of the femur (42%), 2 septic nonunions of the femur (8%), 6 aseptic nonunions of the tibia (25%), 4 septic nonunions of the tibia (17%), and 2 acute post-traumatic femoral bone defects (8%). The cases that were diagnosed with septic nonunion were treated initially with the modified Masquelet technique. The first procedure was debridement and resection of the infected bone, filling the bone defect with cement impregnated with antibiotics (2–4 g of Vancomycin per cement pack). Intravenous antibiotics was administered according to the results of culture. Once the infectious process was controlled, the RIA technique was performed to fill the bone defect.

The volume of the collected graft was measured, as well as the evaluation of postoperative transfusion rate, postoperative pain, and complications (Table 1).

**Table 1:** Pre- and postoperative patient data

Gender	Age	Diagnosis	Transfusion	Preop Hb	Postop Hb	VAS 3 months	VAS 6 months	Graft vol (cc)
F	27	Aseptic nonunion left femur	Not	14,6	10,1	2	1	50
M	34	Aseptic nonunion left femur	1 unit of PRBC + 2 units of plasma	12,6	7,1	2	0	40
M	49	Aseptic nonunion left femur	Not	13,3	9,3	1	0	55
M	50	Aseptic nonunion right femur	1 unit of PRBC	12,9	7,2	1	0	55
M	27	Infected nonunion left tibia	1 unit of PRBC	14,1	7,5	1	0	35
M	30	Infected nonunion left tibia	Not	14,9	9,6	1	0	60
F	27	Infected nonunion right femur	Not	13,6	10,4	1	0	55
M	47	Infected nonunion left tibia	Not	14,7	11,3	2	1	50
M	41	Aseptic nonunion right tibia	Not	13,0	10,4	1	0	45 +
M	33	Aseptic nonunion right femur	Not	14,3	9,7	3	1	50
F	55	Infected nonunion left tibia	1 unit of PRBC	11,2	7,2	1	1	30
F	27	Infected nonunion right femur	Not	14,1	9,4	2	0	50
F	34	Aseptic nonunion left tibia	Not	12,5	10,4	1	0	40
F	49	Aseptic nonunion left femur	Not	12,6	9,6	1	0	45
F	55	Aseptic nonunion left femur	Not	13,2	10,1	1	0	40 +
M	34	Aseptic nonunion left femur	Not	14,2	9,8	1	1	60
M	30	Aseptic nonunion right tibia	Not	12,3	8,3	3	1	50
M	27	Open fracture left femur	Not	14,2	9,4	2	0	45
M	47	Aseptic nonunion left femur	Not	14,7	10,1	2	1	40
M	41	Aseptic nonunion left femur	Not	13,1	9,3	4	2	50
M	36	Aseptic nonunion left femur	Not	13,7	10,4	2	0	45
M	44	Aseptic nonunion right tibia	Not	12,5	9,8	1	0	40
M	27	Aseptic nonunion left tibia	Not	14,3	9,5	1	0	55
F	34	Open fracture left femur	Not	13,2	8,8	2	1	60

+ ICBG (iliac crest bone graft)

*PRBC* Packed red blood cells

*F* Female, *M* Male

**Fig. 2:**
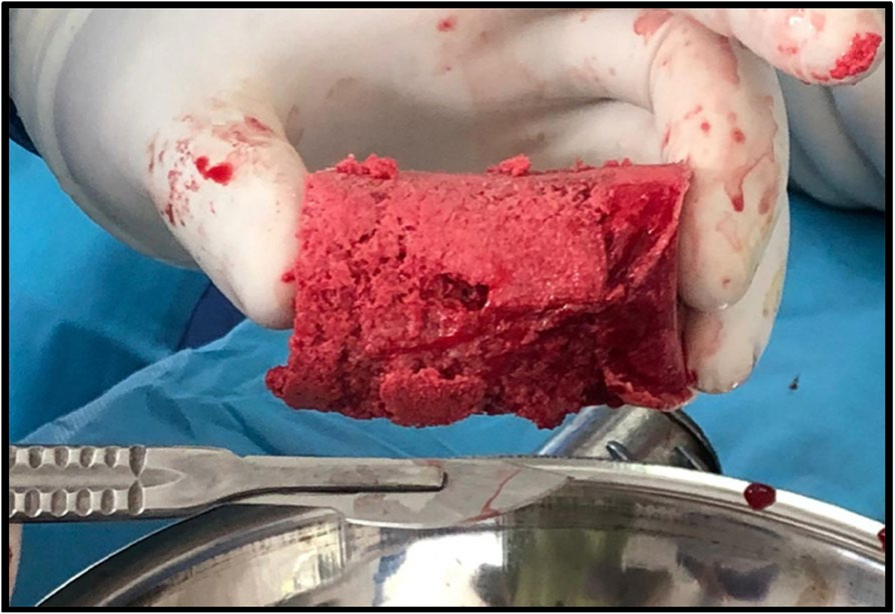
Intraoperative photograph showing the harvested bone graft using the RIA system

**Fig. 3:**
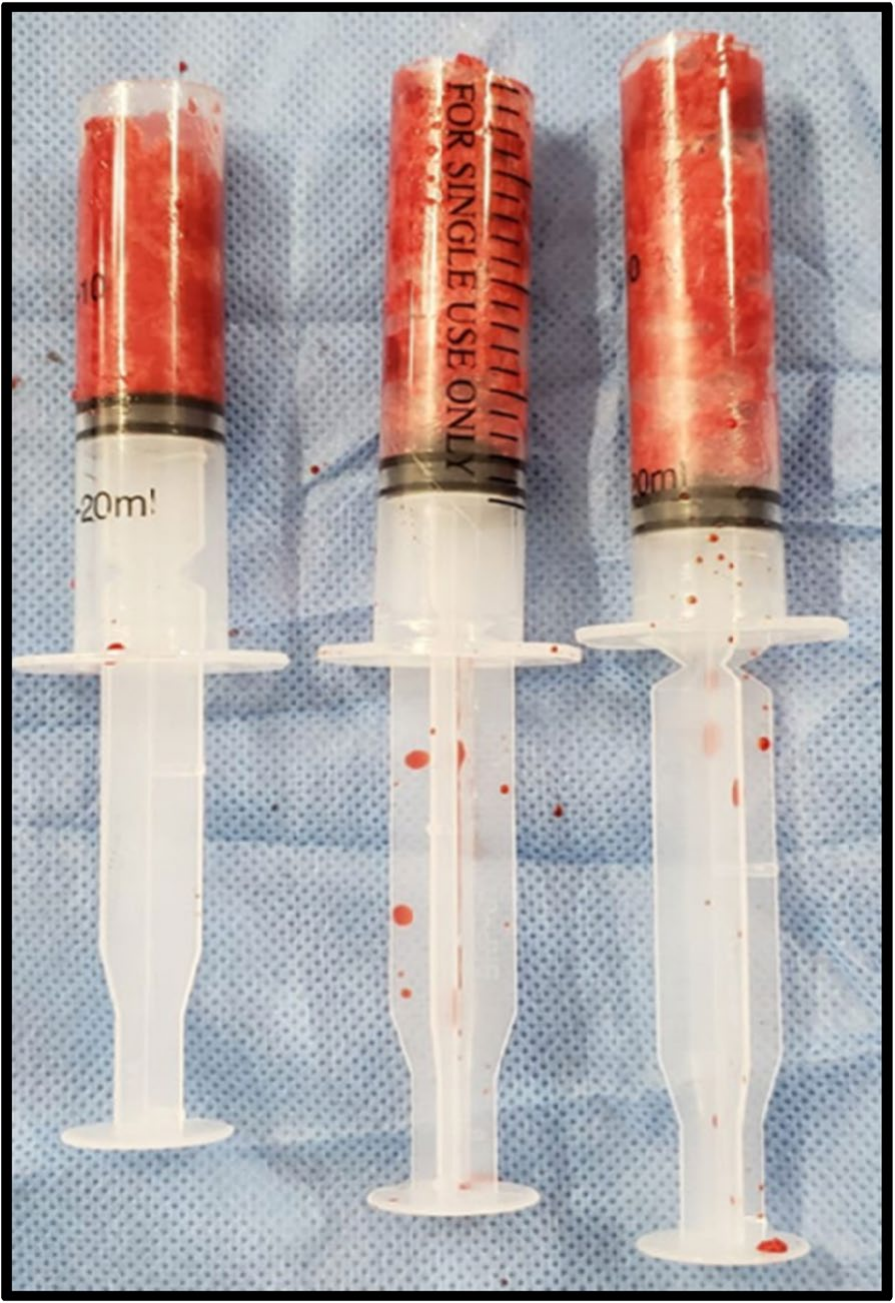
Measurement of the volume of the harvested bone graft using 20 cc syringes

To determine the appropriate size of the reamer, the diameter of the isthmus in the anteroposterior view was measured with a ruler provided by the manufacturer of the RIA system, under fluoroscopy. The reamer diameter was 1.5 mm diameter larger than the isthmus measurement. The volume of collected bone graft was measured in 20 cc syringes (Figs. 2 and 3). At 24 h postoperatively, hemoglobin levels were compared with the preoperative levels. Patients requiring a blood transfusion were registered. The transfusion criteria were: symptomatic patients, hemoglobin less than 8 g/dl or 9 g/dl, in patients with a cardiac history. Regarding postoperative pain, the patients were evaluated at 3 and 6 months. At each visit, the presence or absence of pain was documented using the Visual Analogue Pain Scale (VAS). The present study was approved by the ethics committee of the main institution.

## Results

The bone graft volume was 45 cc on average (range 30–60 cc). In 10 patients (83%), bone grafting by RIA was sufficient to fill the bone defect, while in 2 cases (17%) it was necessary to add autologous iliac crest bone graft to completely fill the bone defect. A mean drop in postoperative hemoglobin of 4.1 g / dL (range 0.5–6.0 g / dL) was evidenced. In 4 cases (33%), a unit of packed red blood cells was required. Regarding postoperative knee pain, average VAS at 3 months was (1.6) and (0.4) at 6 months. No patients reported persistent and significant donor site pain at the time of the last follow-up. No cases of intra- or postoperative femoral fracture were observed.

However, we detected uneccentric reaming in the postoperative radiograph of one patient. In this case, since the femur was ipsilateral to the tibial bone defect, the patient was not allowed to weight bearing until the 12th postoperative week. There were no additional postoperative complications such as infection, heterotopic ossification, and thromboembolic events.

## Discussion

The advantageous biological properties of the harvested bone graft, besides the amount of volume obtained, ratify the RIA system as a safe and efficient alternative to the traditional iliac crest bone grafting [1–5].

Kanakaris et al. reported an average bone graft volume using the RIA system of 65 cc (range 40 to 85 cc) [7]. McCall et al. reported an average volume of 64 ml, being 67 cc from the femur and 37.5 cc from the tibia. The authors highlighted that collected bone graft was not the maximum available volume, since patients with smaller defects needed less bone graft [8]. These results are also comparable with those presented by Han et al., who obtained an average volume of 40.6 cc (range 20 to 80 cc) [9]. In our series, the average volume collected was 45 cc (range 30 to 60 cc). In 10 of the 12 cases, the volume obtained was sufficient to fill the defect, while in the remaining two cases it was supplemented with an autologous iliac crest graft. On the other hand, Stafford et al. present an average volume of 47 cc, requiring the use of complementary material in 23 of their 27 cases [10].

The biological properties of the bone graft using the RIA system were extensively reported on the literature [11, 12]. Sagi et al. evaluated the quantitative and qualitative differences of the bone graft obtained from the medullary canal and the iliac crest, showing that the graft obtained from the iliac crest has clear disadvantages in terms of the morbidity of the donor site and the limited volume obtained. The authors pointed out that the RIA samples had a higher number of mesenchymal stem cells than the iliac crest bone graft, and would also have greater regenerative characteristics [13].

El-Jawhari et al. evaluated the biological fitness of bone progenitor cells in RIA waste. The authors found that the RIA-W bag, which is generally wasted in routine practice may offer great clinical value as an abundant source of functionally competent bone progenitor cells [14].

Due to the possibility of generating eccentric or excessive reaming of the canal during the RIA technique, iatrogenic femoral fractures were previously reported on the literature [6, 9, 15]. Han et al. evaluated 57 patients treated using the RIA system and reported a femoral fracture at the graft harvesting site that required fixation with an intramedullary nail [9]. Belthur et al. also described 2 cases of perforations of the anterior cortex of the femur among their 41 patients treated with RIA [15]. Davis et al., comparing retrograde versus antegrade route using the RIA system in the femur, reported a significant increase in the risk of an iatrogenic fracture of the femur with the antegrade route [6]. Although we detected uneccentric reaming in the postoperative radiograph of one of our patients, there was no postoperative femoral fracture until the last follow-up. Schmitz et al., in a biomechanical cadaveric study, evaluated the influence of the Reamer-Irrigator-Aspirator diameter on femoral bone strength and amount of harvested bone graft. The authors concluded that reaming with RIA diameter of 4.0 mm larger than the femoral isthmus may considerably influence its torsional stiffness, thereby increasing the risk of iatrogenic fracture [16].

Due to the abundant intramedullary blood supply, the presence of hemorrhage is another possible complication when using the RIA technique [1, 2, 17]. During conventional reaming, 100 cc of blood loss may be found [1]. Prolonged suction and aspiration using the RIA system may enhance the blood loss. Han et al. described that 7 of their 57 patients required transfusion after surgery due to a mean drop in hemoglobin of 3.15 g/dl [9]. Marchand et al. conducted a comparative study between the RIA system and the iliac crest autologous bone graft technique and analyzed the estimated amount of blood loss as well as the percentages of blood transfusion. They reported an average loss of 674 cc in the RIA group vs 255 cc in the iliac crest graft group. At the same time, 44% of the patients in the RIA group required a blood transfusion vs 21% in the iliac crest bone graft group [18]. Although in our series we did not directly measure the volume of blood loss, indirectly the average drop in hemoglobin (4.1 g/dl) and the transfusion percentage of 33% are in line with those reported series and highlight the importance of predicting this possibility.

Regarding postoperative pain in the donor area, different studies highlighted lower morbidity associated with RIA compared to autologous iliac crest graft. Belthur et al. conducted a comparative study of patients treated using RIA technique (41 cases) and patients treated with iliac crest bone graft (40 cases), in which a reduction in postoperative pain was found in patients treated with RIA [15]. Davis et al., in his analysis of postoperative pain after the use of the RIA according to the access route, reported greater knee pain in the retrograde group vs greater hip pain in the antegrade group. However, at the end of follow-up, none of the patients manifested discomfort or persistent pain around the knee or the hip. Corroborating with previous reports, we found no persistent pain at the final follow-up in any of the patients.

The study imitations include the retrospective observational study design, the selection bias related to the inclusion criteria, the lack of a control group with the antegrade route and with the traditional technique of autologous bone grafting from the iliac crest, and lack of long-term follow-up to evaluate knee pain, septic arthritis, hemarthrosis, and other complications related to the retrograde approach.

## Conclusion

In this limited case series, the use of the RIA system through a retrograde route in the femur allowed to obtain large volumes of bone graft. There were no intra-/ postoperative complications observed at 6-month follow-up. Therefore this novel technique appears safe and efficacious. Further studies with large sample and prospective design comparing the antegrade and the retrograde pathways for bone grafting using the RIA system are required to fully validate the insights from this pilot study.

## Data Availability

The datasets used and/or analyzed during the current study are available from the corresponding author on reasonable request.

## Abbreviations

RIA: Reamer–Irrigator–Aspirator
VAS: Visual Analogue Pain Scale
PRBC: Packed red blood cells
ICBG: Iliac crest bone graft
